# Sensitivity of Dermatophytes to Terbinafine: World Experience and Recent Findings from Kazakhstan

**DOI:** 10.3390/antibiotics15030266

**Published:** 2026-03-04

**Authors:** Alma Aimoldina, Ainura Smagulova, Yelena Kukhar, Gulnar Batpenova, Togzhan Algazina, Rabiga Uakhit, Vladimir Kiyan

**Affiliations:** 1 Department of Dermatovenereology and Dermatocosmetology, Astana Medical University, Astana 010011, Kazakhstan; aimoldina.a@amu.kz (A.A.); batpenova.g@amu.kz (G.B.); khabdina.t@amu.kz (T.A.); 2 Laboratory of Biodiversity and Genetic Resources, National Center for Biotechnology, Astana 010011, Kazakhstan; smagulova0114@gmail.com (A.S.); erken.uakhitrabiga@gmail.com (R.U.); 3 Research Platform of Agricultural Biotechnology, S. Seifullin Kazakh Agrotechnical Research University, Astana 010011, Kazakhstan; kucharev@mail.ru; 4 Scientific Center for Biological Research, Astana 010011, Kazakhstan

**Keywords:** *Trichophyton* spp., *Microsporum* spp., terbinafine, sensitivity, EUCAST, dermatophyte

## Abstract

**Background/Objectives**: This article describes the results of an analysis of the sensitivity of dermatophytosis pathogens to terbinafine, conducted by the authors based on a review of available scientific publications and data from their own research. Currently, no information is available on the sensitivity of Kazakh isolates obtained from patients at dermatological clinics. The aim of this study was to compile data on the resistance of dermatophytes to terbinafine over the past decade worldwide and investigate the sensitivity of dermatophyte isolates collected from patients in Astana, Kazakhstan, to terbinafine. **Methods**: A systematic review and meta-analysis were conducted following the Preferred Reporting Items for Systematic Reviews and Meta-Analysis (PRISMA) guidelines, utilizing the Pubmed and Cochrane Library databases with specific keywords. The sensitivity of the dermatophytes to terbinafine was assessed using EUCAST E.Def 11.0 method. **Results**: Screening of terbinafine susceptibility among Kazakh clinical isolates revealed that all *Microsporum canis* strains (57/57, 100%) were sensitive to the drug. Among 33 *Trichophyton* spp. isolates, 4 (12.1%) demonstrated resistance to terbinafine, with MIC values ranging from 0.125 to 1.5 µg/mL. The resistant isolates belonged to the species *T. indotineae*, *T. interdigitale*, and *T. mentagrophytes*. **Conclusions**: Terbinafine remains highly effective against *Microsporum canis* in Kazakhstan, while a small proportion of *Trichophyton* isolates show resistance. Continuous monitoring of dermatophyte susceptibility is warranted to guide effective treatment.

## 1. Introduction

Dermatophytes are microscopic fungi that feed on keratinized tissues. Some species have adapted to parasitism in animals and humans and have become obligate pathogens [1]. The primary pathogens causing human dermatophytosis belong to the genera *Trichophyton*, *Microsporum* and *Epidermophyton*. The introduction of third-generation systemic antimycotics in the 1990s (in particular, terbinafine and itraconazole) largely solved the problem of effective and safe therapy for dermatophytosis [2]. However, resistant clinical strains have been reported from 2017 onward [3,4]. As of 2021, 17 of 20 European countries covered by the questionnaire of the mycological working group of the European Academy of Dermatology and Venereology (EADV) have registered cases of clinical and microbiological resistance in skin dermatophytosis, that is, *Trichophyton* and *Microsporum* infections [5].

The effectiveness of dermatophytosis treatment depends on several factors. These include the clinical form and prevalence of the disease, degree of damage to the skin appendages, presence of concomitant diseases, and appointment by the doctor of a full-fledged treatment regimen and its observance by the patient [6]. The development of atypical, generalized, and antifungal-resistant forms of dermatomycosis can be facilitated by immune system disorders, both congenital, including mutations in the CARD9 gene [7,8], and acquired, such as those arising from the therapy of immune-dependent dermatoses with corticosteroid hormones or immunobiological drugs [9,10,11,12].

A decrease in the effectiveness of antifungal drugs has been observed under both laboratory conditions and in clinical practice. With a decrease in the effectiveness of the antifungal agent *in vitro*, this can be said about the true (or microbiological) resistance of the fungus [13]. Although treatment failure is not always caused by antifungal resistance, since factors such as inadequate dosing, poor patient compliance, or pharmacokinetic variability may also contribute, antimicrobial resistance remains a significant and well-documented phenomenon that helps to assess the extent of reduced antifungal efficacy. Epidemiological studies conducted in different countries have revealed increased minimum inhibitory concentrations (MICs) of terbinafine against clinical *T. interdigitale* [14,15] and *T. rubrum* [16]. In the treatment of dermatomycosis caused by *Microsporum canis*, the share of therapeutic failures was 25–40%. Their causes, along with the resistance of the pathogen, could be low patient compliance, insufficient penetration of the antifungal drug into the affected tissues, and varying bioavailability of the drug, which is also true for failure in the treatment of other dermatophyte infections [5,17,18,19].

Thus, existing data indicate the clinical and microbiological resistance of dermatophytes globally. Resistance amongst *Trichophyton* spp. is most commonly reported in India and Iran. Considering the ongoing dynamics of pathogenic fungal sensitivity, the research findings published in the past 10 years remain relevant. These findings will help to objectively assess the global state of dermatophyte resistance to antifungal drugs as comprehensively as possible. However, it is worth noting that there are no meta-analytical studies that would allow us to systematize these data.

The primary purpose of this study was to systematize data on the resistance of dermatophytes to terbinafine over the past 10 years and to study the sensitivity of dermatophyte isolates collected from patients in Astana (Kazakhstan).

## 2. Results

### 2.1. Study the Prevalence of Dermatophytes Sensitivity to Terbinafine in Global Practice

A total of 4828 dermatophyte isolates were studied for sensitivity to terbinafine in 21 studies conducted between 2014 and 2023 that met the selection criteria. The largest number of studies was conducted in India (n = 6) and Iran (n = 5); two articles were published by authors from Japan, and one publication each was from Denmark, France, Germany, Pakistan, Greece, China and Belgium. In one study, isolates from India, China, Australia, Germany, and the Netherlands were tested [20].

Antimycotic sensitivity has been investigated in most publications using the micro-broth dilution method of the Clinical Laboratory Standards Institute (CLSI) and European Committee on Antimicro fungistatic bial Susceptibility Testing (EUCAST). In the analyzed studies, the resistance to terbinafine was studied among the following spectrum of dermatophytic fungi: *T. rubrum*, *T. mentagrophytes*, *T. interdigitale*, *T. violaceum*, *T. tonsurans*, *M. canis*, *T. verrucosum*, *M. gypseum*, *A. vanbreuseghemii*, *T. soudanense*, *Epidermophyton floccosum*, *N. gypsea*, *N. fulva*, *T. benhamiae*, *M. audouinii*, *N. incurvata*.

The country, sample size, prevalence of terbinafine resistance, and design of the 21 selected studies are presented in Table 1.

As shown in Table 1, among terbinafine-resistant dermatophytes, the species *T. mentagrophytes* and *T. indotineae* prevailed. The incidence of terbinafine-resistant dermatophytes was 0–81.8%. The highest percentages of terbinafine-resistant dermatophytes were observed in studies conducted on Indian isolates, with rates of 81.8% and 61.0%, respectively [30,35]. The resistance of dermatophytic fungi to antifungal drugs has not been reported by authors from other countries [28,37,38].

The minimum inhibitory concentration of terbinafine (MIC) in the analyzed studies ranged from 0.001 to ≥32 µg/mL. The highest MIC value (≥32 µg/mL) was noted in 2 studies for *T. interdigitale* and *T. mentagrophytes*, as well as for *T. indotineae*,* T. mentagrophytes*/*T. interdigitale complex* and *T. rubrum*—one study for each fungus. All six studies that reported the highest MIC values for terbinafine were conducted in India and Iran.

### 2.2. Clinical and Etiological Characteristics of Dermatomycoses

An analysis of data from ten studies, which indicated clinical forms of dermatomycosis caused by a certain type of fungus, showed that in total, 1992 isolates of dermatophytes were studied. The relationship between the clinical forms of dermatophytosis and pathogens is shown in Table 2.

As shown in Table 2, tinea corporis was predominantly caused by *T. interdigitale* and *T. mentagrophytes*, tinea cruris was primarily caused by *E. floccosum*, onychomycosis was primarily attributed to *T. rubrum*, and *T. interdigitale* was the predominant species responsible for tinea pedis. Among the analyzed studies, two articles were devoted to only one clinical form, tinea capitis, in which the leading pathogens were *T. violaceum* and *M. audouinii*.

### 2.3. Demography and Clinical Details (Own Research)

In total, 91 male and female patients with an established diagnosis of dermatophytosis were examined. Male patients were predominant, accounting for 64.83% (n = 59) of all the patients. The ages of the participants were within the range of 2–83 years (the average age was 17.4 years). The following distribution was observed according to the clinical form: 49 patients (53.85%) had dermatophytosis of the smooth skin, 33 patients (36.26%) were diagnosed with ringworm of the scalp, and 9 patients (9.89%) with ringworm of the groin area. More than half of the patients (53.7%, n = 44) reported contact with stray or domestic cats as a potential source of infection.

### 2.4. Antifungal Susceptibility Testing

Between February and December 2023, a total of 90 clinical isolates from Kazakhstan (Astana) were included in this study (Table 3). According to the morphological (macro- and microscopic) characteristics of the colonies and the localization of clinical lesions, 33 isolates were identified as *Trichophyton* spp. (36.7%) and 57 belonged to the *Microsporum canis* (63.3%). One isolate was identified as *Candida* sp. and, as it did not belong to the target group of dermatophytes, it was excluded from further analysis and not included in the study sample.

**Figure 1:**
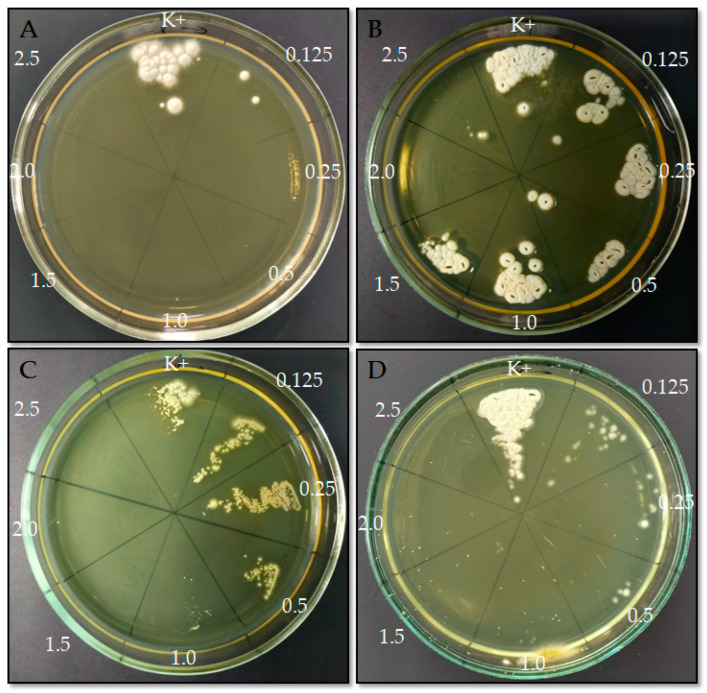
Culture results of dermatomycosis strains following terbinafine susceptibility testing using the EUCAST method: (**A**) *T. mentagrophytes*, (**B**) *T. tonsurans*, (**C**), *T. indotineae*, (**D**) *T. interdigitale*. K+ indicates the well without terbinafine; numbers indicate terbinafine concentrations (µg/mL).

Initial screening of the 90 isolates for terbinafine resistance was performed using the EUCAST E.Def 11.0 method. A total of 4 out of 90 isolates (4.4%) showed viability, as evidenced by growth on Sabouraud medium after exposure to terbinafine (Figure 1).

One resistant isolate was identified as *T. tonsurans* (n = 1) based on morphological and genetic characteristics, resulting in a relative resistance frequency of 11.1% (1/9). One resistant isolate was identified as *T. interdigitale* (n = 1), with a relative resistance frequency of 33.3% (1/3). Two additional resistant isolates were identified as *T. mentagrophytes* (n = 2) based on morphological characteristics and clinical localization (Table 4). Molecular analysis revealed that one of the two resistant isolates was *T. indotineae*.

The EUCAST method for microconidia-forming dermatophytes was used to determine the antifungal susceptibility to terbinafine of 90 isolates (16 *T. mentagrophytes*, 9 *T. tonsurans*, 4 *T. verrucosum*, 3 *T. interdigitale*, 1 *T. indotineae*, and 57 *M. canis*). As described above (Table 3), two isolates exhibited terbinafine MIC values of 1 and 1.5 µg/mL, corresponding to relative resistance frequencies of 100% (1/1) and 33.3% (1/3) for *T. indotineae* and *T. interdigitale*, respectively. For the other species, terbinafine MIC values were below 1.5 µg/mL and were 0.125 µg/mL for *T. mentagrophytes* and 0.5 µg/mL for *T. interdigitale*.

## 3. Discussion

Terbinafine is an antifungal agent administered orally and topically. Terbinafine hydrochloride is a synthetic derivative of allylamine, a white or almost white fine crystalline powder that is easily soluble in methanol and methylene chloride, soluble in ethanol, and slightly soluble in water, with a molecular weight of 327.90. Chemical Formula: ((E)-N-(6,6-Dimethyl-2-heptene-4-ynyl)-N-methyl-1-naphthalene methanamine) [40].

The spectrum of *in vitro* activity includes a wide range of dermatophytes, filamentous, dimorphic, and dematic fungi, as well as some types of yeast. Terbinafine has a positive effect on various strains of dermatophytes, such as *E. floccosum*, *Malassezia furfur*, *M. canis*, *M. gypseum*, *M. nanum*, *T. mentagrophytes*, *T. rubrum* and *T. verrucosum* [41]; therefore, it is widely used in the treatment of various skin dermatophytes. The minimum inhibitory concentration for *T. rubrum* is in the range of 0.001–0.06 µg/mL [42,43].

Dermatophytes of the genus *Trichophyton* are sensitive to most antifungal drugs both *in vitro* and *in vivo*. The resistance of dermatophytes to terbinafine is also known [4,23,33,44].

In the treatment of dermatomycosis caused by *M. canis*, the cause of failures (25–40%) may be the high resistance of the pathogen, low patient compliance, insufficient penetration of the antifungal drug into the affected tissues, and varying bioavailability of the drug [17,18,19].

Terbinafine exerts a fungicidal effect by inhibiting squalene monooxygenase, which is involved in sterol synthesis in fungi. This inhibits fungal sterol biosynthesis by reducing ergosterol levels. The formation and growth of fungal cell membranes are disrupted because ergosterol is one of the primary components of the cell membrane [40].

Reports of increasing resistance to terbinafine in various countries, including Europe [5], India [45], Iceland [46], and Iran [44], have prompted studies examining the level of resistance of local Kazakhstani strains isolated from patients in dermatological clinics.

*Trichophyton* spp. strains showed both pronounced sensitivity (87.9%; *T. mentagrophytes*—15 strains, *T. verrucosum*—4 strains, *T. tonsurans*—8 strains, and *T. interdigitale*—2 strains) and resistance (12.1%; *T. mentagrophytes*—1 strain, *T. indotineae*—1 strain, *T. tonsurans*—1 strain, and *T. interdigitale*—1 strain) to terbinafine. Studies on the sensitivity of strains to terbinafine *in vitro* conducted against *Trichophyton* spp. and *Microsporum canis* strains indicated high sensitivity to antimycotics in Kazakh strains. This is consistent with data from studies conducted on dermatophyte isolates from China [28], Japan [38], and Iran [34,37].

*M. canis* strains (n = 57) demonstrated high susceptibility to terbinafine, with an MIC of 0.125 µg/mL. Sabouraud agar cultures obtained after EUCAST terbinafine susceptibility testing showed no growth of *M. canis* at any tested terbinafine concentration, whereas growth was observed in the control wells. These findings are consistent with previously published data. In studies by Zeeshan et al. (2023) [25] and Pashootan et al. (2022) [27], *M. canis* likewise exhibited high susceptibility to terbinafine, with reported MIC values of 0.05 (0.28) µg/mL and 0.03–0.125 µg/mL, respectively.

In the present study, terbinafine susceptibility testing revealed variable MIC values among *Trichophyton* species. Two isolates exhibited elevated MICs of 1 and 1.5 µg/mL, corresponding to relative resistance frequencies of 100% (1/1) for *T. indotineae* and 33.3% (1/3) for *T. interdigitale*. In contrast, the remaining isolates demonstrated lower MIC values (<1.5 µg/mL), with *T. mentagrophytes* and *T. interdigitale* showing MICs of 0.125 µg/mL and 0.5 µg/mL, respectively. These findings indicate that, while most *Trichophyton* isolates from Kazakhstan remain susceptible to terbinafine, resistant strains are already present, particularly among *T. indotineae* and *T. interdigitale*.

Our results for *T. mentagrophytes* are consistent with previously published data, which generally report high susceptibility to terbinafine. For instance, Moreno-Sabater et al. (2022) [15] reported an MIC of 0.03 µg/mL, Siopi et al. (2021) [26] reported an MIC of 0.127 µg/mL, and Behnam et al. (2020) [34] found MICs ranging from 0.002 to 1 µg/mL. These comparable low MIC values support the notion that *T. mentagrophytes* remains largely sensitive to terbinafine in different geographic regions.

In contrast, *T. indotineae* has been frequently associated with terbinafine resistance in multiple studies. Although this species was not prevalent among our isolates, the resistant strain identified in our study aligns with the global trend of emerging terbinafine resistance. Several reports have documented high MIC values for *T. indotineae*, including 2–4 µg/mL in Astvad et al. (2022) [21], 0.015–32 µg/mL in Pashootan et al. (2022) [27], and 0.016–16 µg/mL in Kong et al. (2021) [20]. This pattern highlights the potential for rapid spread of resistant *T. indotineae* strains and underscores the need for ongoing surveillance and antifungal susceptibility testing in clinical practice.

In recent years, terbinafine resistance in dermatophytes has been assessed not only by MIC testing but also by molecular detection of resistance-associated mutations in the squalene epoxidase gene (SQLE), the molecular target of terbinafine [4,20,32]. This genotypic approach enables rapid screening for known target-site alterations, particularly in regions with a high prevalence of terbinafine-resistant strains within the *T. mentagrophytes*/*T. interdigitale complex* and *T. indotineae*. Nevertheless, SQLE genotyping cannot fully substitute for phenotypic susceptibility testing, because MIC values reflect the overall susceptibility phenotype and may also be influenced by additional or yet unidentified mechanisms beyond target-site changes. Therefore, we relied on the EUCAST reference method E.Def 11.0, which provides standardized and reproducible MIC determination, to assess susceptibility among our clinical isolates from Astana. In our collection, most isolates remained susceptible, although several *Trichophyton isolates* demonstrated elevated MIC values (up to 1.5 µg/mL). A limitation of the present study is that SQLE mutations were not investigated in these isolates; thus, future surveillance in Kazakhstan should ideally combine EUCAST MIC testing with SQLE sequencing (or targeted mutation assays) to strengthen genotype–phenotype correlation and facilitate early detection of resistant strains.

Overall, our data suggest that terbinafine remains effective against most dermatophyte isolates in Kazakhstan, but the emergence of resistant *T. indotineae* and *T. interdigitale* strains warrants attention. Continuous monitoring of antifungal susceptibility, combined with molecular characterization of resistant isolates, is essential to guide appropriate treatment strategies and prevent further dissemination of resistant strains.

## 4. Materials and Methods

### 4.1. An Ethics Statements

The literature review was based on previously published studies; therefore, ethical approval or patient consent was not required. Our own research with the participation of people was reviewed and approved by the local ethics committee of the Astana Medical University NpJSC (decision No. 5, meeting No. 2 dated 23 February 2023). Each participant or legal guardian signed a written informed consent from to participate in the study.

### 4.2. Search Strategy

This systematic review and meta-analysis was conducted in accordance with the recommendations of the Preferred Reporting Items for Systematic Reviews and Meta-Analysis (PRISMA). Before the quantitative and systematic synthesis, we examined the results of studies aimed at assessing the prevalence of terbinafine resistance in dermatophytic fungi.

**Figure 2:**
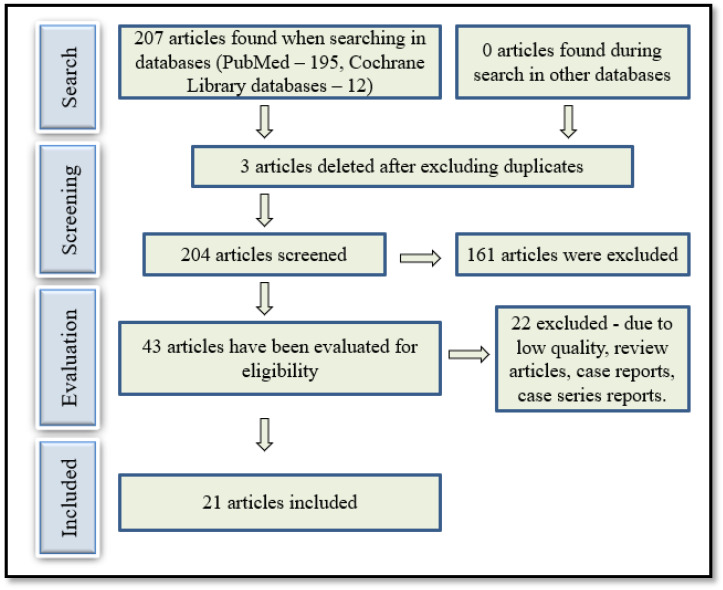
Literature review scheme and article selection criteria.

A comprehensive database search was performed using PubMed and the Cochrane Library. During the initial literature search in the mentioned databases, keywords such as “terbinafine”, “sensitivity” and “resistance” were used. The following search criteria were used for PubMed (Ovid MEDLINE): [“Dermatophytes” (MeSH)] AND [“Terbinafine” (MeSH) and “Sensitivity” (MeSH) OR “Resistance” (MeSH) (title/abstract)]. The search was limited to articles published in English and Russian between 2013 and 2024. Subsequently, we evaluated the abstracts and titles of all the identified articles to determine whether they met the inclusion criteria. Finally, we reviewed the reference lists of all relevant articles to identify additional relevant studies (Figure 2).

### 4.3. Inclusion Criteria

The inclusion criteria were as follows: (i) studies that included patients with dermatophytosis; (ii) studies that evaluated the sensitivity to terbinafine; and (iii) studies published in English.

### 4.4. Exclusion Criteria

The exclusion criteria were as follows: (i) studies that did not indicate the frequency of resistance and/or MIC to terbinafine among patients with dermatophytosis; (ii) lack of full text for full review; (iii) studies with low methodological quality, that is, case reports, case series and comments; and (iv) studies published in languages other than English.

### 4.5. Selection of Articles

The initial search and selection of articles were performed independently by several co-authors who checked the titles and abstracts and excluded all articles that did not meet the inclusion criteria. Next, we received the full texts of the articles that were deemed appropriate and evaluated all studies based on their designs. Any disagreements regarding inclusion were resolved through discussion. The selection process based on the PRISMA recommendations is shown in Figure 2.

Studies provided sufficient data, such as a quantitative assessment of the prevalence of dermatophyte resistance to terbinafine and a comparison of the minimum inhibitory concentration (MIC) of terbinafine in the studied cultures.

### 4.6. Data Extraction and Evaluation of the Study

Data were extracted from articles corresponding to the search criteria and presented in a standardized form, which contained: (i) the surname of the first author and the year of publication; (ii) the country of origin of the study; (iii) the sample size of patient groups; (iv) the prevalence of terbinafine resistance in dermatophytic fungi; and (v) indicators of terbinafine MIC, if any. The quality of the included articles was independently evaluated, after which they were collectively agreed upon.

### 4.7. Patient Inclusion Criteria (Own Research)

This study included 91 patients of both sexes with an established diagnosis of dermatophytosis who visited the dermatological unit of City Multidisciplinary Hospital No. 3 for Astana (Kazakhstan) between February and December 2023. Patients with nail lesions alone were excluded. Disease history, life history, and epidemiological background data were also collected. Therefore, we conducted an objective clinical examination of patients with dermatophytosis.

### 4.8. Methods for Culture and Identification

Samples of biological material (hair and scales) were collected from the affected area of the skin on day 0 by scraping, after proper skin antisepsis of the affected area with 70% alcohol, and delivered to the mycological laboratory. The samples were sown on Sabouraud chloramphenicol (50 mg/L) dextrose agar (Condalab, Madrid, Spain). During the initial isolation of the pathogen (10 days) and upon receipt of a pure culture, surface cultivation of the fungus was carried out at a temperature of 28 °C for at least 6 days until the formation of characteristic colonies. Mycological diagnosis was made 10 days later. The types of pathogens were determined based on cultural and morphological characteristics of the colonies, and microscopic morphology. Polymerase chain reaction (PCR) was performed to identify the genetic diversity of strains using 1 primer pair targeting ribosomal RNA genes: forward *ITS1* (5′-TCCGTAGGTGAACCTGCGG-3′) and reverse *ITS4* (5′-TCCTCCGCTTATTGATATGC-3′) [39]. The PCR-amplified target gene fragment was purified using a Quick PCR Purification Kit (Invitrogen, Vilnius, Lithuania) following the manufacturer’s protocol. Sequencing was performed using a Seq Studio Genetic Analyzer (Thermo Fisher Scientific Applied Biosystems, Waltham, MA, USA) according to the manufacturer’s instructions. The resulting nucleotide sequences were visually checked using BioCapt software (version 11.0). Nucleotide sequences of the studied species were compared with other sequences in the NCBI GenBank database using BLAST. The antifungal susceptibility of isolated pure cultures of the pathogen was assessed with terbinafine.

### 4.9. Antifungal Susceptibility Testing

Screening of clinical isolates was performed using the reference EUCAST method E.Def 11.0, as previously described [47]. The isolates were subcultured on Sabouraud chloramphenicol (50 mg/L) dextrose agar (Condalab, Madrid, Spain) and incubated at 27 °C for 4–7 days. Inoculum suspensions were prepared from fresh, mature cultures by washing with sterile water. The suspension was vortexed for 15 s using a vortex mixer (Eppendorf, Hamburg, Germany) at 2000 rpm and then transferred into a sterile syringe fitted with a sterile filter (pore size 11 µm), filtered, and collected in a sterile tube. The suspension was adjusted with sterile distilled water to a concentration of 1 × 10^6^ microconidia/mL by counting the microconidia in a hemocytometer chamber (Merck, S.A., Madrid, Spain). Then, the optical density at 530 nm (OD530) of the suspensions was measured in a spectrophotometer (Eppendorf, Hamburg, Germany). The colonies were counted as soon as possible after the observation of visible growth. The suspensions were then diluted 1:10 with sterile distilled water to obtain a final working inoculum of 1 × 10^5^ CFU/mL [48,49]. Each well of the microdilution plate was inoculated with 100 µL of the 1 × 10^5^ CFU/mL microconidial suspension. Strains were tested using a range of terbinafine concentrations (0.125–5.0 mg/L), beginning with the EUCAST-recommended MIC of 0.125 mg/L [50]. Growth control wells, containing 100 µL of sterile drug-free medium, were also inoculated with 100 µL of the same inoculum suspension. Microdilution plates were incubated without agitation at 27 °C in ambient air. Results were read daily from day 1 to day 6 of incubation.

The inhibitory effect of terbinafine on all clinical fungal isolates was evaluated by subculturing the contents of the wells onto Sabouraud chloramphenicol (50 mg/L) dextrose agar (Condalab, Madrid, Spain). Fungal growth was assessed visually after 10 days.

### 4.10. Statistical Analysis

Statistical analyses were performed using SPSS version 15 (SPSS Inc., Chicago, IL, USA) for Windows using one-factor analysis of variance. If necessary, descriptive statistics were used, as well as the chi-square and the Fisher–Mann–Whitney U-criterion. Statistical significance was set at *p* < 0.05.

## 5. Conclusions

Differences in dermatophyte sensitivity to terbinafine across regions and countries can be attributed to various factors, including genetic variability among fungal strains, environmental conditions, variations in antifungal drug usage practices, and host factors. These factors can affect the following aspects:

1. Genetic variability. Fungal populations can exhibit genetic diversity due to various factors such as mutations, genetic recombination, and migration. Different strains of dermatophytes may have different degrees of sensitivity to antifungal drugs such as terbinafine. Because of their nature, strains common in Kazakhstan may exhibit a higher sensitivity to terbinafine than strains common in other regions.
2. Features of the antifungal drugs. Therefore, the frequency and duration of antifungal drug use may affect the development of drug resistance. If terbinafine is used widely or incorrectly in certain regions, it can exert selective pressure on dermatophyte populations, contributing to the emergence of resistant strains. Conversely, if the use of terbinafine is limited or reasonably controlled, it may contribute to the preservation of sensitivity among dermatophyte isolates.
3. Environmental factors. Environmental conditions such as temperature, humidity, and geographic location can affect the prevalence and characteristics of fungal populations. Certain conditions can promote the growth and spread of certain strains of dermatophytes that are sensitive to terbinafine. Differences in environmental conditions between Kazakhstan and other countries may contribute to the differences in drug sensitivity profiles.
4. Host factors. Host factors, including immune status, genetic predisposition, and the anatomical site of infection, can also influence the response of dermatophytic infections to antifungal treatment. The host factors prevailing in the studied population in Astana (Kazakhstan) may contribute to the higher sensitivity of dermatophytes to terbinafine compared with that of populations in other countries.

Thus, the susceptibility of dermatophytes to terbinafine may vary by region owing to a combination of genetic, environmental, and host-related factors.

## Figures and Tables

**Table 1:** Country, sample size, and prevalence of terbinafine resistance and design of 21 studies.

First Author, Year	A Country	Sample Size	Number of Cases of Terbinafine Resistance	Prevalence of Resistance (%)	MIC of Terbinafine (µg/mL)	Method for Determining Sensitivity
Astvad, 2022 [21]	Denmark	63	35	55.6	*T. rubrum* ≤0.004–>4; *T. indotineae* 2–>4; *T. mentagrophytes*/*T. interdigitale* ≤0.004–0.016	EUCAST reference method E.Def 11.0
Moreno-Sabater, 2022 [15]	France	580	3	0.23/1.1/16.7	*T. indotineae* 0.008–2; *T. interdigitale* 0.008–8; *T. mentagrophytes* 0.03	EUCAST microdilution broth method
Shankarnarayan, 2020 [22]	India	146	15	10.3	*T. mentagrophytes* 2–16 mg/L	Micro-broth dilution technique as per CLSI M38 A2 protocol
Yamada, 2017 [23]	Japan	2056	17	1	*T. interdigitale*—0.00625; *A. vanbreuseghemii*—0.00625–3.2; *T. rubrum*—0.00625–3.2	Broth microdilution method of the CLSI
Nenoff, 2020 [24]	Germany	29	13	45	≥0.2 μg/mL	Broth microdilution method of the CLSI
Zeeshan, 2023 [25]	Pakistan	61	7	11.5	*T. violaceum*—0.16 (0.24), *T. mentagrophytes* 0.19 (0.26), *T. tonsurans* 0.08 (0.09), *T. soudanense* 0.23 (0.32), *T. rubrum* 0.03 (0.13), *T. verrucosum* 0.32 (0.27), *M. canis* 0.05 (0.28), *M. gypseum* 0.14 (0.15)	Broth microdilution method of the CLSI
Taghipour, 2020 [4]	Iran	141	5	3.5	*T. interdigitale*—0.003–0.25, *T. mentagrophytes*—0.007–≥32	Broth microdilution method of the CLSI
Siopi, 2021 [26]	Greece	112	9	8.0	*T. rubrum* 0.022 (≤0.008–0.03), *T. mentagrophytes*—0.127, (≤0.008–8), *T. interdigitale*—0.013 (≤0.008–0.03), *T. tonsurans* 0.016 (0.016–0.016)	EUCAST broth microdilution reference methodology (E.DEF 11.0)
Pashootan, 2022 [27]	Iran	123	6	4.9	*T. indotineae*—0.015–32, *T. mentagrophytes*—0.015–16, *T. interdigitale*—0.003–0.025, *T. rubrum*—0.003–0.006, *T. tonsurans*—0.003–0.125, *E. floccosum*—0.06–16, *T. verrucosum*—0.06–4, *N. gypsea*—0.015–16, *N. fulva*—0.125–0.25, *M. canis*—0.03–0.125	Broth microdilution method of the CLSI
Jiang, 2021 [28]	China	62	0	0	0.001–0.015	Broth microdilution method of the CLSI
Bhatia, 2015 [29]	India	53	11	20.8	*T. mentagrophyte*—0.0625–4, *T. rubrum*—0.0313–1, *M. gypseum*—2.0	Broth microdilution method of the CLSI
Khurana, 2018 [30]	India	64	39	61	0.25–≥32	Broth microdilution method of the CLSI
Mohammadifard, 2022 [31]	Iran	24	8	15.1/14.2	0.003–≥32	Broth microdilution method of the CLSI
Sacheli, 2020 [32]	Belgium	337	6	20	0.016–4	EUCAST E.Def 9.3.1 procedure
Rudramurthy, 2018 [33]	India	133	20	15.0	*T. interdigitale*—0.015–32, *T. rubrum*—0.015–16, *T. tonsurans* 0.015–2	Broth microdilution method of the CLSI
Behnam, 2020 [34]	Iran	75	0	0	*T. mentagrophytes*—0.002–1, *T. interdigital*—0.002–1, *T. tonsurans*—0.002–0.5, *E. floccosum*—0.125–1, *M. canis*—0.002–0.125, *N. fulvum*—1, *T. benhamiae*—1, *T. verrucosum*—1, Dermatophyte isolates—0.002–1.	Broth microdilution method of the CLSI
Singh, 2019 [35]	India	44	36	81.8	0.06–16	Broth microdilution method of the CLSI
Shaw, 2020 [36]	India	498	57	11.4	0.015–32	Broth microdilution method of the CLSI
Afshari, 2016 [37]	Iran	49	0	0	0.0313–16	Disk diffusion assay; broth microdilution method of the CLSI
Tamura, 2014 [38]	Japan	43	0	0	*T. rubrum*—0.004–0.06, *T. mentagrophytes*—0.03–0.06, *T. verrucosum*—0.015, *T. tonsurans*—0.015–0.06, *M. canis*—0.008–0.03, *M. gypseum*—0.004–0.06, *E. floccosum*—0.015–0.03	Broth microdilution method of the CLSI
Kong, 2021 [20]	India, China, Australia, Germany, The Netherlands	135	34	25.2	*T. indotineae*—0.016–>16, *T. interdigitale*—0.016–0.0625, *T. mentagrophytes*—0.016–0.0625	EUCAST E.Def 9.3.1 protocol

**Table 2:** Distribution of dermatophytes based on clinical forms and etiological agent.

Dermatophyte	Clinical Form of Dermatomycosis							A Country	First Author, Year
	Tinea Corporis, n (%)	Tinea Capitis, n (%)	Tinea Faciei, n (%)	Tinea Cruris, n (%)	Onychomy-Cosis, n (%)	Tinea Pedis, n (%)	Tinea Manis, n (%)		
*T. rubrum*	7 (1.6)	0 (0.0)	0 (0.0)	24 (5.5)	292 (67.0)	112 (25.7)	1 (0.2)	France	Moreno-Sabater, 2022 [15]
	0 (0.0)	2 (3.3)	0 (0.0)	0 (0.0)	0 (0.0)	0 (0.0)	0 (0.0)	Pakistan	Zeeshan, 2023 [25]
	24 (34.3)	0 (0.0)	0 (0.0)	0 (0.0)	46 (65.7)	0 (0.0)	0 (0.0)	Greece	Siopi, 2021 [26]
	2 (3.2)	1 (1.6)	1 (1.6)	1 (1.6)	20 (32.3)	36 (58.1)	1 (1.6)	China	Jiang, 2021 [28]
	1 (16.7)	0 (0.0)	0 (0.0)	1 (16.7)	0 (0.0)	2 (33.3)	2 (33.3)	Iran	Afshari, 2016 [37]
	0 (0.0)	0 (0.0)	0 (0.0)	5 (33.3)	0 (0.0)	7 (46.7)	3 (20.0)	Iran	Pashootan, 2022 [27]
*T. interdigitale*	96 (70.6)	0 (0.0)	0 (0.0)	35 (25.7)	0 (0.0)	3 (2.2)	2 (1.5)	France	Moreno-Sabater, 2022 [15]
	5 (5.2)	0 (0.0)	0 (0.0)	0 (0.0)	20 (20.8)	71 (74.0)	0 (0.0)	Iran	Taghipour, 2020 [4]
	0 (0.0)	0 (0.0)	0 (0.0)	0 (0.0)	12 (100)	0 (0.0)	0 (0.0)	Greece	Siopi, 2021 [26]
	2 (20)	0 (0.0)	0 (0.0)	0 (0.0)	0 (0.0)	5 (50)	3 (30)	Iran	Afshari, 2016 [37]
	1 (3.6)	2 (7.1)	0 (0.0)	11 (39.3)	0 (0.0)	9 (32.1)	5 (17.9)	Iran	Pashootan, 2022 [27]
*T. mentagrophytes*	2 (25.0)	0 (0.0)	0 (0.0)	0 (0.0)	1 (12.5)	5 (62.5)	0 (0.0)	France	Moreno-Sabater, 2022 [15]
	20 (66.7)	0 (0.0)	2 (6.7)	4 (13.3)	2 (6.7)	1 (3.3)	1 (3.3)	Germany	Nenoff, 2020 [24]
	0 (0.0)	18 (29.5)	0 (0.0)	0 (0.0)	0 (0.0)	0 (0.0)	0 (0.0)	Pakistan	Zeeshan, 2023 [25]
	43 (95.6)	1 (2.2)	0 (0.0)	0 (0.0)	1 (2.2)	0 (0.0)	0 (0.0)	Iran	Taghipour, 2020 [4]
	10 (41.7)	0 (0.0)	0 (0.0)	14 (58.3)	0 (0.0)	0 (0.0)	0 (0.0)	Greece	Siopi, 2021 [26]
	0 (0.0)	5 (1.5)	0 (0.0)	0 (0.0)	0 (0.0)	0 (0.0)	0 (0.0)	Belgium	Sacheli, 2020 [32]
	481 (96.6)	13 (2.6)	0 (0.0)	0 (0.0)	4 (0.8)	0 (0.0)	0 (0.0)	India	Shaw, 2020 [36]
	1 (16.7)	0 (0.0)	0 (0.0)	2 (33.3)	0 (0.0)	2 (33.3)	1 (16.7)	Iran	Pashootan, 2022 [27]
*T. violaceum*	0 (0.0)	21 (34.4)	0 (0.0)	0 (0.0)	0 (0.0)	0 (0.0)	0 (0.0)	Pakistan	Zeeshan, 2023 [25]
	0 (0.0)	28 (8.2)	0 (0.0)	0 (0.0)	0 (0.0)	0 (0.0)	0 (0.0)	Belgium	Sacheli, 2020 [32]
*T. tonsurans*	0 (0.0)	9 (14.8)	0 (0.0)	0 (0.0)	0 (0.0)	0 (0.0)	0 (0.0)	Pakistan	Zeeshan, 2023 [25]
	0 (0.0)	57 (16.8)	0 (0.0)	0 (0.0)	0 (0.0)	0 (0.0)	0 (0.0)	Belgium	Sacheli, 2020 [32]
	2 (29)	2 (29)	1 (14)	1 (14)	0 (0.0)	1 (14)	0 (0.0)	Iran	Afshari, 2016 [37]
	2 (12.5)	8 (50.0)	3 (18.75)	3 (18.75)	0 (0.0)	0 (0.0)	0 (0.0)	Iran	Pashootan, 2022 [27]
*T. indotineae*	2 (20.0)	0 (0.0)	0 (0.0)	5 (50.0)	0 (0.0)	3 (30.0)	0 (0.0)	Iran	Pashootan, 2022 [27]
*T. soudanense*	0 (0.0)	3 (4.9)	0 (0.0)	0 (0.0)	0 (0.0)	0 (0.0)	0 (0.0)	Pakistan	Zeeshan, 2023 [25]
*T. verrucosum*	0 (0.0)	3 (4.9)	0 (0.0)	0 (0.0)	0 (0.0)	0 (0.0)	0 (0.0)	Pakistan	Zeeshan, 2023 [25]
	1 (16.7)	0 (0.0)	1 (16.7)	0 (0.0)	2 (33.3)	1 (16.7)	1 (16.7)	Iran	Afshari, 2016 [37]
	0 (0.0)	0 (0.0)	5 (71.4)	0 (0.0)	0 (0.0)	0 (0.0)	2 (28.6)	Iran	Pashootan, 2022 [27]
*M. canis*	0 (0.0)	3 (4.9)	0 (0.0)	0 (0.0)	0 (0.0)	0 (0.0)	0 (0.0)	Pakistan	Zeeshan, 2023 [25]
	0 (0.0)	36 (10.6)	0 (0.0)	0 (0.0)	0 (0.0)	0 (0.0)	0 (0.0)	Belgium	Sacheli, 2020 [32]
	6 (100)	0 (0.0)	0 (0.0)	0 (0.0)	0 (0.0)	0 (0.0)	0 (0.0)	Iran	Afshari, 2016 [37]
	5 (35.7)	4 (28.6)	0 (0.0)	1 (7.1)	0 (0.0)	0 (0.0)	4 (28.6)	Iran	Pashootan, 2022 [27]
*M. gypseum*	0 (0.0)	2 (3.3)	0 (0.0)	0 (0.0)	0 (0.0)	0 (0.0)	0 (0.0)	Pakistan	Zeeshan, 2023 [25]
	2 (40)	0 (0.0)	0 (0.0)	1 (20)	0 (0.0)	2 (40)	0 (0.0)	Iran	Afshari, 2016 [37]
*M. audouinii*	0 (0.0)	120 (35.4)	0 (0.0)	0 (0.0)	0 (0.0)	0 (0.0)	0 (0.0)	Belgium	Sacheli, 2020 [32]
*T. soudanense*	0 (0.0)	85 (25.1)	0 (0.0)	0 (0.0)	0 (0.0)	0 (0.0)	0 (0.0)	Belgium	Sacheli, 2020 [32]
*T. benhamiae*	0 (0.0)	7 (2.1)	0 (0.0)	0 (0.0)	0 (0.0)	0 (0.0)	0 (0.0)	Belgium	Sacheli, 2020 [32]
*N. incurvata*	0 (0.0)	1 (0.2)	0 (0.0)	0 (0.0)	0 (0.0)	0 (0.0)	0 (0.0)	Belgium	Sacheli, 2020 [32]
*E. floccosum*	2 (22.2)	0 (0.0)	0 (0.0)	5 (55.6)	0 (0.0)	2 (22.2)	0 (0.0)	Iran	Afshari, 2016 [37]
	1 (5.3)	0 (0.0)	0 (0.0)	14 (73.7)	0 (0.0)	3 (15.7)	1 (5.3)	Iran	Pashootan, 2022 [27]
*N. gypsea*	0 (0.0)	2 (33.3)	0 (0.0)	0 (0.0)	4 (66.7)	0 (0.0)	0 (0.0)	Iran	Pashootan, 2022 [27]
*N. fulva*	0 (0.0)	0 (0.0)	0 (0.0)	0 (0.0)	2 (100.0)	0 (0.0)	0 (0.0)	Iran	Pashootan, 2022 [27]

**Table 3:** Data on 90 isolates from Kazakhstan recovered from patients, identified based on lesion morphology and clinical localization.

Identification by Conventional Methods	Clinical Forms of Dermatophytosis		
Type of Pathogen	Tinea Cruris n (%)	Tinea Corporis n (%)	Tinea Capitits n (%)
*T. mentagrophytes* (n = 16)	6 (37.5)	8 (50)	2 (12.5)
*T. verrucosum* (n = 4)	1 (25)	2 (50)	1 (25)
*T. tonsurans* (n = 9)		5 (55.5)	4 (44.4)
*T. indotineae* (n = 1)	1 (100)		
*T. interdigitale* (3)	1 (33.3)	2 (66.6)	
*M. canis* (57)		31 (54.38)	26 (45.61)

**Table 4:** Antifungal susceptibility and molecular characteristics of strain isolates and patient information.

Identification		Antifungal Susceptibility MIC (µg/mL)	Patient Information	
Classical Methods	Molecular Methods [39]	Terbinafine	Patient (n), Origin/Travel	Clinical Form
*T. mentagrophytes*	*T. mentagrophytes*	0.125	1, Kazakhstan, Astana	Tinea corporis
*T. tonsurans*	*T. tonsurans*	1.5	1, Kazakhstan, Astana	Tinea corporis
*T. mentagrophytes*	*T. indotineae*	1.0	1, Kazakhstan, Astana	Tinea cruris
*T. interdigitale*	*T. interdigitale*	0.5	1, Kazakhstan, Astana	Tinea corporis

## Data Availability

Data are contained within the article.
